# Phenotypic and transcriptomic characterization of two newly described Mycobacterium species within the *Mycobacterium gordonae* complex

**DOI:** 10.3389/fmicb.2026.1810237

**Published:** 2026-05-20

**Authors:** Tian Gan, Youming Mei, Haiqin Jiang, Wenyue Zhang, Ying Shi, Hongsheng Wang

**Affiliations:** 1 Hospital for Skin Disease, Institute of Dermatology, Chinese Academy of Medical Sciences and Peking Union Medical College, Nanjing, China; 2 Jiangsu Key Laboratory of Molecular Biology for Skin Diseases and STIs, Nanjing, China

**Keywords:** immunometabolism, macrophage, *Mycobacterium gordonae* complex, non-tuberculous mycobacteria, transcriptome

## Abstract

**Introduction:**

Non-tuberculous mycobacteria constitute a rapidly emerging class of opportunistic pathogens with escalating clinical significance globally. We recently characterized two novel species within the Mycobacterium gordonae complex-Mycobacterium camsae and Mycobacterium pumcae-both isolated from human cutaneous infections.

**Methods:**

Comprehensive phenotypic and transcriptomic analyses were conducted to elucidate their pathobiological properties. Comparative analysis included *M. gordonae* (closest phylogenetic relative), *M. marinum* (slow-growing reference), and *M. abscessus* (rapid-growing reference). Growth kinetics, intracellular survival, cytokine induction, and bacterial and host transcriptomic profiling were characterized.

**Results:**

Both novel species exhibited attenuated growth kinetics characteristic of the *M. gordonae* complex. Notably, *M. pumcae* demonstrated enhanced inflammatory potential resembling *M. marinum*, while *M. camsae* displayed more subdued host responses akin to *M. gordonae*. Bacterial transcriptomic profiling unveiled distinct adaptive strategies: *M. pumcae* preferentially upregulated stress response regulons and iron homeostasis pathways, whereas *M. camsae* emphasized cell envelope biosynthesis and core metabolic reconfiguration. Host transcriptomic analysis at 24 h post-infection revealed differential temporal dynamics: the rapid-growing *M. abscessus* predominantly triggered immunometabolic reprogramming pathways, while slow-growing mycobacteria (*M. camsae, M. pumcae*, and *M. gordonae*) were still eliciting robust innate immune recognition signatures.

**Discussion:**

This investigation provides the first systematic characterization of host-pathogen interactions within these newly described *M. gordonae* complex members, establishing a mechanistic foundation for understanding their distinct pathogenic trajectories and informing species-specific diagnostic and therapeutic approaches.

## 1 Introduction

Non-tuberculous mycobacteria (NTM) are ubiquitous environmental organisms and opportunistic pathogens capable of causing diverse pulmonary and extrapulmonary diseases (Griffith et al., 2007; Desai and Hurtado, 2021). The *Mycobacterium gordonae* complex represents one of the most prevalent NTM populations in aquatic environments (Tortoli, 2014). While traditionally regarded as environmental contaminants with minimal pathogenic potential, members of this complex have been increasingly recognized as causative agents of clinically significant infections (Chang et al., 2021; Chen et al., 2017). Given that many *M. gordonae* complex organisms share high 16S rRNA gene identity, genome-based taxonomic approaches have facilitated the recognition of new species.

We recently described two novel species within the *M. gordonae* complex: *Mycobacterium camsae* sp. nov. X7091^T^ and *Mycobacterium pumcae* sp. nov. Z3061^T^, both isolated from human cutaneous infections in Jiangsu Province, China (Mei et al., 2025). While the taxonomic and baseline phenotypic descriptions were established, the pathogenic mechanisms and host immune responses elicited by these species remain largely unexplored.

Understanding mycobacteria-host interactions is fundamental to elucidating pathogenic mechanisms and developing therapeutic strategies (Bo et al., 2023). Macrophages are key cellular targets for mycobacterial infection and represent the frontline of innate immune defense (Hmama et al., 2015). Upon mycobacterial infection, macrophages activate pattern recognition receptor (PRR) signaling pathways, triggering the production of pro-inflammatory cytokines such as tumor necrosis factor-alpha (TNF-α), interleukin-6 (IL-6), and interleukin-1 beta (IL-1β). These cytokines play essential roles in bacterial containment and adaptive immune activation (Roca et al., 2019; Hamilton et al., 2025; Di Paolo et al., 2015). Simultaneously, intracellular mycobacteria reprogram host cellular metabolism, particularly lipid and glucose metabolism, to establish favorable replicative niches (Russell, 2001; Hashish et al., 2018).

In this study, we performed comprehensive phenotypic and transcriptomic characterization of *M. camsae* and *M. pumcae* alongside their phylogenetically close relative *M. gordonae*, as well as two clinically important NTM species representing distinct growth rates: the rapidly growing *M. abscessus* and the slowly growing *M. marinum*. We characterized growth kinetics, intracellular survival, cytokine induction profiles, and performed transcriptomic analyses of both bacterial cultures and infected host cells. Our findings reveal distinct pathogenic signatures for these novel species and provide mechanistic insights into their pathogenic potential and clinical significance within the *M. gordonae* complex.

## 2 Results

### 2.1 Phylogenetic positioning and growth characteristics

**Figure 1:**
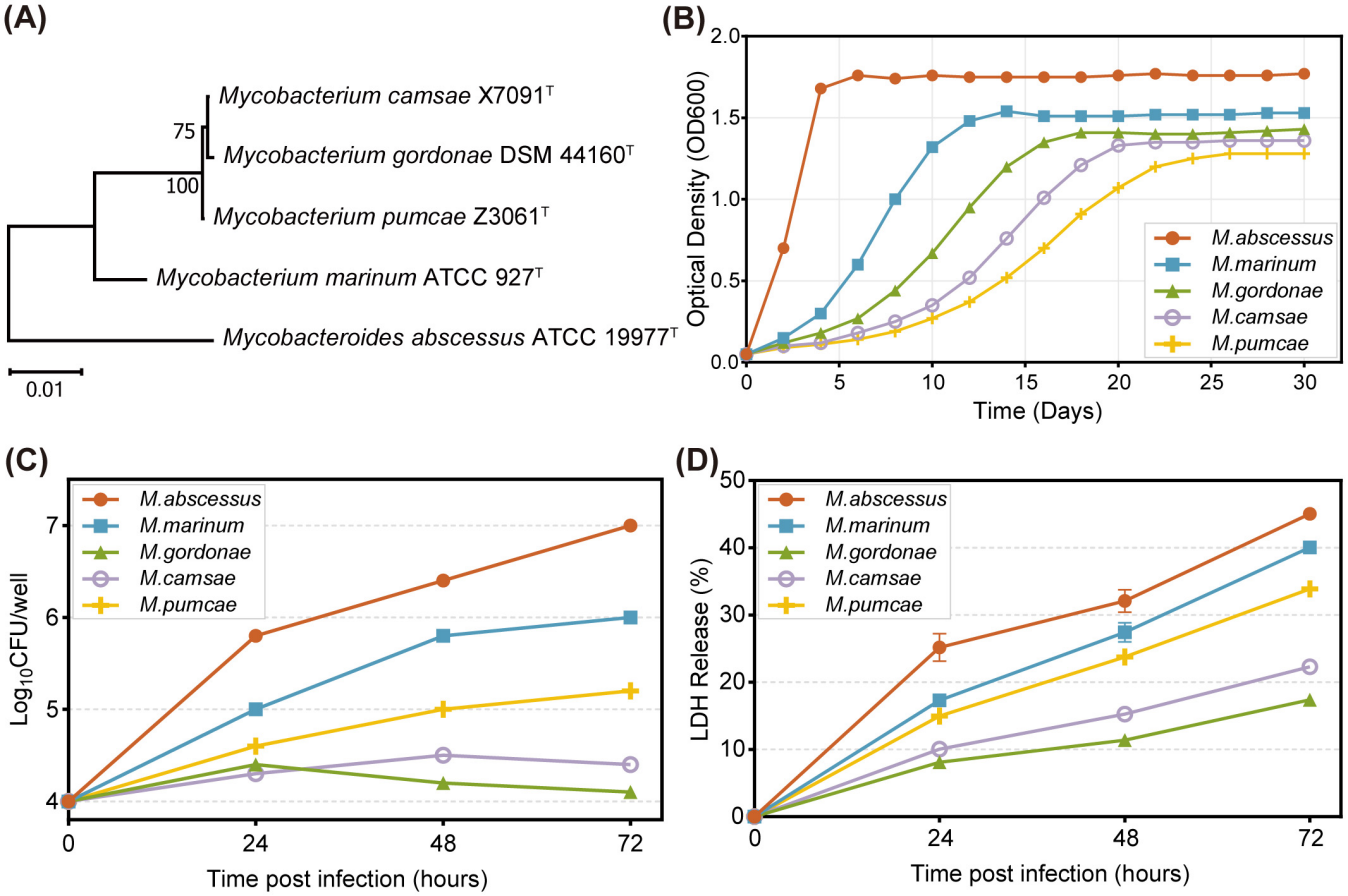
Phylogenetic relationships, growth characteristics, and host responses to mycobacterial strains. **(A)** Neighbor-joining phylogenetic tree based on 16S rRNA gene sequences showing the evolutionary relationships among the five mycobacterial strains used in this study. The scale bar represents 0.01 nucleotide substitutions per site. **(B)** Growth curves of five mycobacterial strains in Middlebrook 7H9 liquid medium. OD_600_ was monitored over 30 days. **(C)** Intracellular survival and replication of mycobacterial strains in THP-1–derived macrophages at an MOI of 10:1. Bacterial loads were determined by CFU enumeration at 0, 24, 48, and 72 hpi. Data are shown as log_10_ CFU per well (mean ± SEM, *n* = 3). **(D)** LDH release assay showing macrophage cytotoxicity at 0, 24, 48, and 72 hpi. Data are expressed as percentage of maximum LDH release (mean ± SEM, *n* = 3). Statistical significance was assessed by one-way ANOVA with Tukey's post hoc test at each time point. *Mab, M. abscessus*; *Mma, M. marinum*; *Mgo, M. gordonae*; *Mca, M. camsae*; *Mpu, M. pumcae*.

We first constructed a phylogenetic tree based on 16S rRNA gene sequences of the five mycobacterial strains (Figure 1A). Consistent with our previous genomic analyses (Mei et al., 2025), *M. camsae* and *M. pumcae* clustered within the *M. gordonae* complex, forming a subclade within the slow-growing mycobacteria (SGM).

We next examined the axenic growth characteristics of all five species in Middlebrook 7H9 broth (Figure 1B). Both novel species exhibited typical slow-growing profiles, with doubling times significantly longer than *M. abscessus*. Notably, *M. pumcae* showed the slowest growth rate among the tested strains, reaching stationary phase later than *M. camsae* and *M. gordonae*.

### 2.2 Intracellular persistence and growth in THP-1-derived macrophages

We further evaluated the ability of these novel species to survive and replicate within THP-1-derived macrophages. *M. abscessus* (rapid-grower) and *M. marinum* (slow-grower) served as high-persistence references, both showing steady increases in intracellular CFU over 72 h (Figure 1C). The novel species and *M. gordonae* exhibited divergent intracellular fates. *M. gordonae* was effectively controlled by macrophages, with CFU counts declining after 24 h. Similarly, *M. camsae* showed transient persistence but was largely cleared by the host cells after 48 h. In contrast, *M. pumcae* demonstrated a slow but continuous intracellular increase from 24 to 72 h, maintaining a higher bacterial load than *M. camsae* and *M. gordonae*, which more closely resembled the persistence pattern of *M. marinum*. It should be noted that for *M. abscessus*, the increasing CFU counts at 48–72 hpi may reflect a combination of intracellular replication and extracellular bacterial proliferation following macrophage lysis, as confirmed by the significant LDH release at these time points (Figure 1D).

### 2.3 Pro-inflammatory cytokine responses to mycobacterial infection

**Figure 2:**
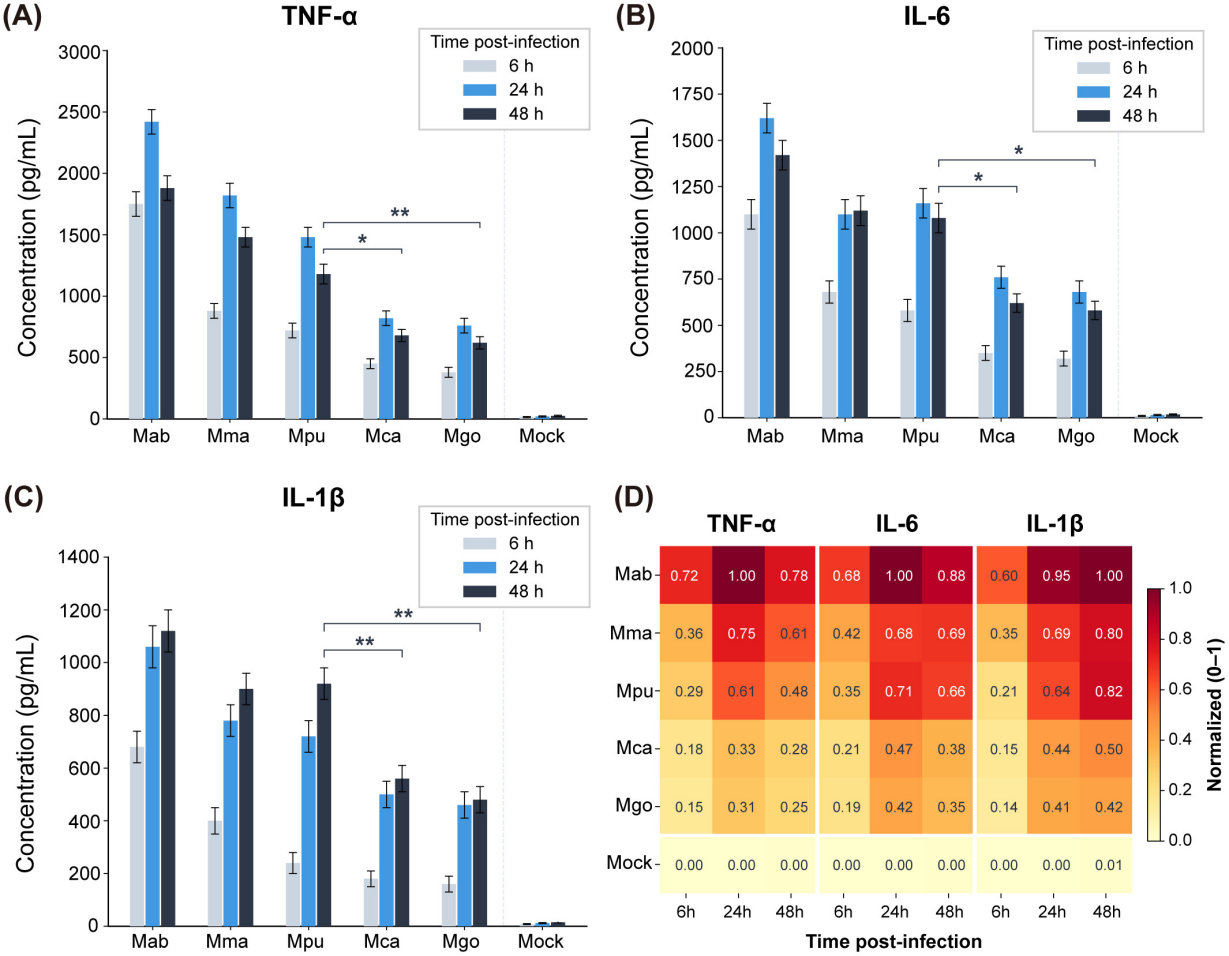
Cytokine production by THP-1-derived macrophages following mycobacterial infection. **(A–C)** Concentrations of TNF-α **(A)**, IL-6 **(B)**, and IL-1β **(C)** in culture supernatants at 6, 24, and 48 hpi, measured by ELISA. Data represent mean ± SEM from three independent experiments performed in duplicate. Statistical significance was assessed by two-way ANOVA with Tukey's post hoc test. *p < 0.05; **p < 0.01. Selected biologically relevant comparisons (*M. pumcae* vs. *M. camsae* and *M. pumcae* vs. *M. gordonae* at 48 hpi) are annotated. **(D)** Min-Max normalized heatmap showing relative cytokine levels across all six groups for three cytokines at three time points. Normalization was performed independently for each cytokine (0 = minimum, 1 = maximum). *Mab, M. abscessus; Mma, M. marinum; Mpu, M. pumcae; Mca, M. camsae; Mgo, M. gordonae*.

To characterize the host response, we measured the secretion of TNF-α, IL-6, and IL-1β at 6, 24, and 48 hpi (Figures 2A–D). *M. abscessus* consistently induced the highest levels of all three cytokines across all time points, reflecting the robust inflammatory response typically elicited by rapid-growing mycobacteria (RGM). *M. marinum* also induced strong cytokine production. Among the *M. gordonae* complex members, *M. pumcae* was a significantly stronger inducer of pro-inflammatory cytokines than *M. camsae* and *M. gordonae*. Notably, by 48 h, *M. pumcae* maintained high levels of IL-1β and TNF-α comparable to *M. marinum*, whereas the cytokine response to *M. camsae* and *M. gordonae* remained relatively low.

### 2.4 Prokaryotic transcriptome profiling of *M. camsae* and *M. pumcae*

**Figure 3:**
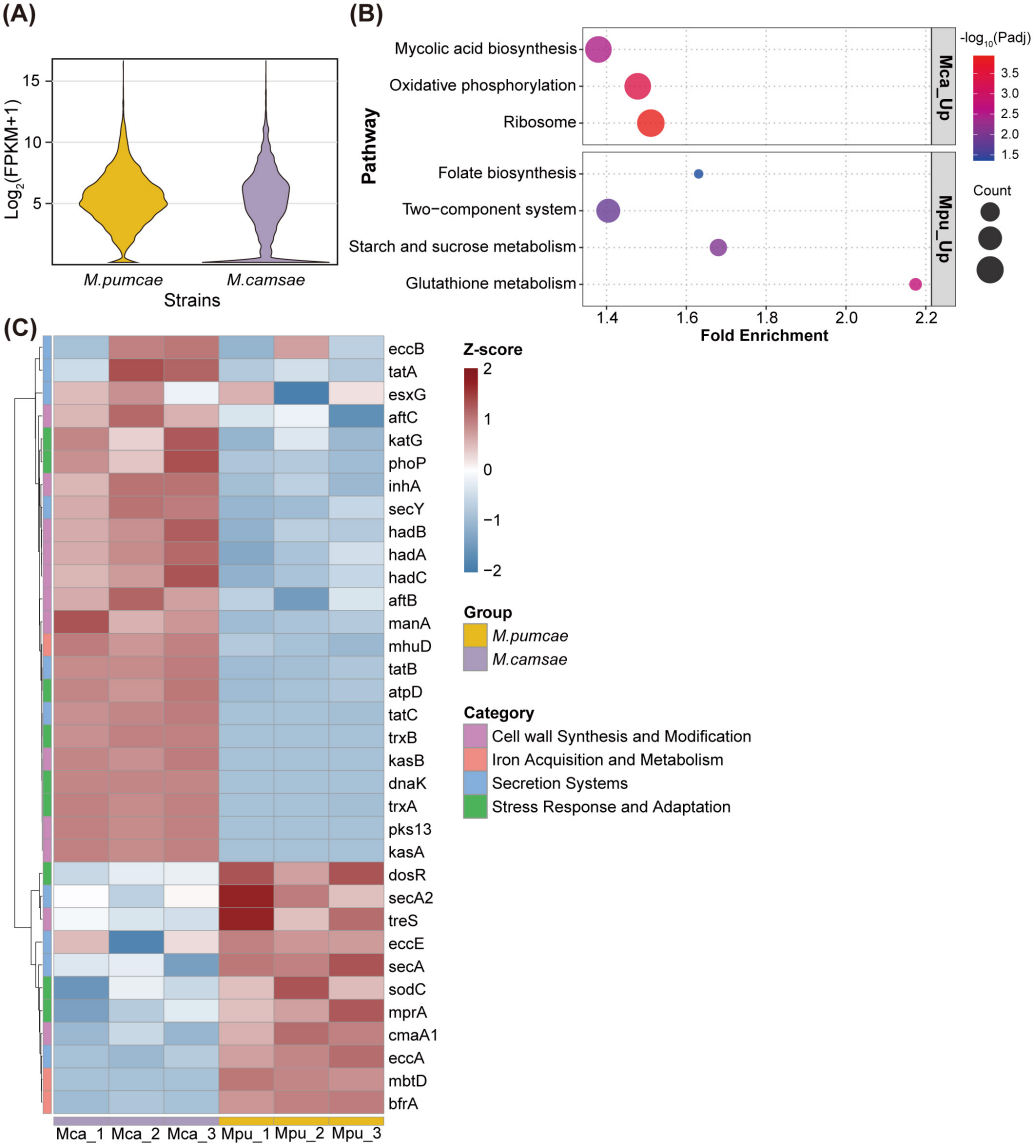
Comparative transcriptomic profiling of *M. camsae* and *M. pumcae* under axenic culture conditions. **(A)** Violin plots showing the overall distribution of gene expression levels in *M. camsae* and *M. pumcae*. Gene expression is presented as log_2_ (FPKM + 1). **(B)** KEGG pathway enrichment analysis of differentially expressed genes between *M. pumcae* and *M. camsae*. Bubble size represents the number of genes in each pathway; color indicates -log10(Padj). Pathways are separated by regulation direction (Mca_up: genes upregulated in *M. camsae*; Mpu_up: genes upregulated in *M. pumcae*). **(C)** Heatmap showing the expression profiles of key genes involved in bacterial virulence and survival across four functional categories: cell wall synthesis and modification (purple), iron acquisition and metabolism (pink), secretion systems (blue), and stress response and adaptation (green). Expression levels are displayed as *z*-scores calculated from log_2_ (FPKM + 1) values, with red indicating higher expression and blue indicating lower expression. Each column represents an individual biological replicate (Mca_1–3, *M. camsae*; Mpu_1–3, *M. pumcae*). Genes were clustered using hierarchical clustering with Euclidean distance.

To investigate the molecular basis underlying the phenotypic differences between the two novel species, we performed RNA seq on *M. camsae* and *M. pumcae* during exponential growth in Middlebrook 7H9 broth. Gene expression profiling revealed broadly similar overall transcriptomic landscapes between the two species. However, the violin plot indicates that *M. camsae* contains a larger fraction of lowly expressed genes, suggesting a more quiescent transcriptional state under these conditions compared to *M. pumcae* (Figure 3A).

KEGG enrichment analysis identified distinct metabolic and regulatory priorities (Figure 3B). In *M. camsae*, upregulated genes were significantly enriched in core biosynthetic and energy-related pathways, including mycolic acid biosynthesis, oxidative phosphorylation, and ribosomal components. This enrichment suggests a prioritized investment in maintaining cell wall integrity and basic translational machinery during axenic growth. Conversely, *M. pumcae* showed enrichment in two-component systems, starch and sucrose metabolism, and glutathione metabolism, indicating a potentially more robust capacity for environmental sensing and managing oxidative stress.

We examined the expression profiles of genes involved in key functional categories: secretion systems, cell wall synthesis and modification, iron acquisition and metabolism, and stress response and adaptation (Figure 3C). Hierarchical clustering revealed distinct expression patterns between the two species across these functional categories. Notably, *M. camsae* exhibited higher expression of several genes involved in cell wall biosynthesis, including *hadA, hadB, hadC* (encoding 3-hydroxyacyl-ACP dehydratases involved in mycolic acid biosynthesis), and *kasA, kasB* (encoding β-ketoacyl-ACP synthases). In contrast, *M. pumcae* demonstrated significantly higher levels of stress-related regulators, most notably the dormancy survival regulator *dosR* and *mprA*, as well as the antioxidant enzyme *sodC*. Furthermore, iron storage and acquisition genes, including *bfrA* and *mbtD*, were more highly expressed in *M. pumcae*. This elevated expression of stress adaptation and iron management modules in *M. pumcae* aligns with its superior intracellular persistence observed in infection assays, suggesting that *M. pumcae* is transcriptionally better equipped to survive the hostile environment within host macrophages.

### 2.5 Host transcriptomic responses and differential gene expression across mycobacterial infections

**Figure 4:**
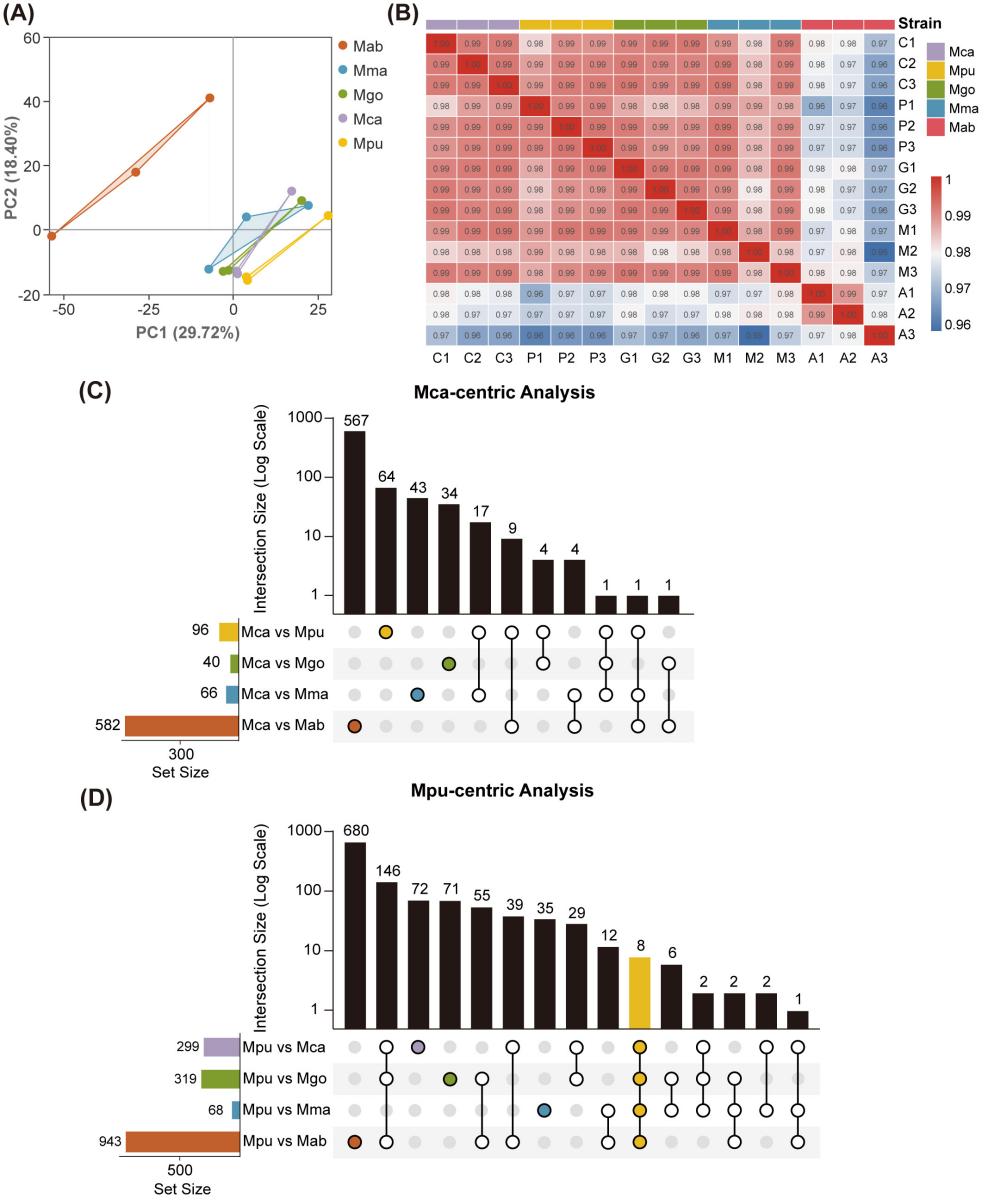
Host transcriptomic analysis of macrophages infected with five mycobacterial species. **(A)** PCA of host gene expression profiles. Each point represents one biological replicate, and colors indicate different strains. PC1 and PC2 explain 29.72% and 18.40% of the total variance, respectively. **(B)** Pearson correlation heatmap of all 15 host transcriptome samples. Sample IDs: C1–C3 (*M. camsae*), P1–P3 (*M. pumcae*), G1–G3 (*M. gordonae*), M1–M3 (*M. marinum*), A1–A3 (*M. abscessus*). **(C, D)** UpSet plots showing intersections of upregulated differentially expressed genes (DEGs) for *M. camsae* **(C)** and *M. pumcae* **(D)** compared to the other four species. For exploratory visualization of global DEG overlap patterns, genes with raw p-value < 0.05 and |log_2_FC| > 1 are shown. In each plot, the horizontal bar chart on the left shows the total number of upregulated DEGs in each pairwise comparison (set size). The dot matrix indicates which comparisons contribute to each intersection, and the vertical bar chart on the top shows the size of each intersection (log scale). *Mab, M. abscessus*; *Mma, M. marinum*; *Mpu, M. pumcae*; *Mca, M. camsae*; *Mgo, M. gordonae*.

We performed RNA-seq on THP-1-derived macrophages at 24 hpi to characterize host transcriptional responses across mycobacterial species. Principal component analysis (PCA) revealed clear species-dependent clustering: *M. abscessus* formed a distinct cluster separated from all slow-growing infections, indicating a markedly different host response to this rapid-growing species. In contrast, macrophages infected with *M. gordonae, M. camsae, M. pumcae*, and *M. marinum* grouped more closely (Figure 4A). Pearson correlation analysis further confirmed high intra-group reproducibility (*r* > 0.97) and highlighted that the host responses to *M. camsae, M. pumcae*, and *M. gordonae* were the most transcriptionally similar among all tested groups (Figure 4B).

To resolve host genes uniquely or sharedly responsive to the novel species, we performed pairwise differential expression analyses focusing on upregulated differentially expressed genes (DEGs) (Figures 4C, D). In both *M. camsae*- and *M. pumcae*-centric analyses, the largest cohorts of upregulated genes were identified in comparisons against the rapid-grower *M. abscessus* (582 and 943 genes, respectively), with the vast majority being unique to these specific contrasts. This underscores a profound divergence in host transcriptional engagement between the novel slow-growers and the rapid-growing pathogen. While host responses among the slow-growing group were more similar, *M. pumcae* exhibited higher divergence than *M. camsae* when compared to other members of the *M. gordonae* complex, yielding 299 upregulated genes relative to *M. camsae* and 319 relative to *M. gordonae*. This broader repertoire of upregulated host genes aligns with the superior intracellular persistence and more robust pro-inflammatory potential of *M. pumcae*, suggesting it triggers a more extensive host defensive program than *M. camsae*.

### 2.6 Immune recognition and metabolic reprogramming define the host response contrast at 24 hpi

**Figure 5:**
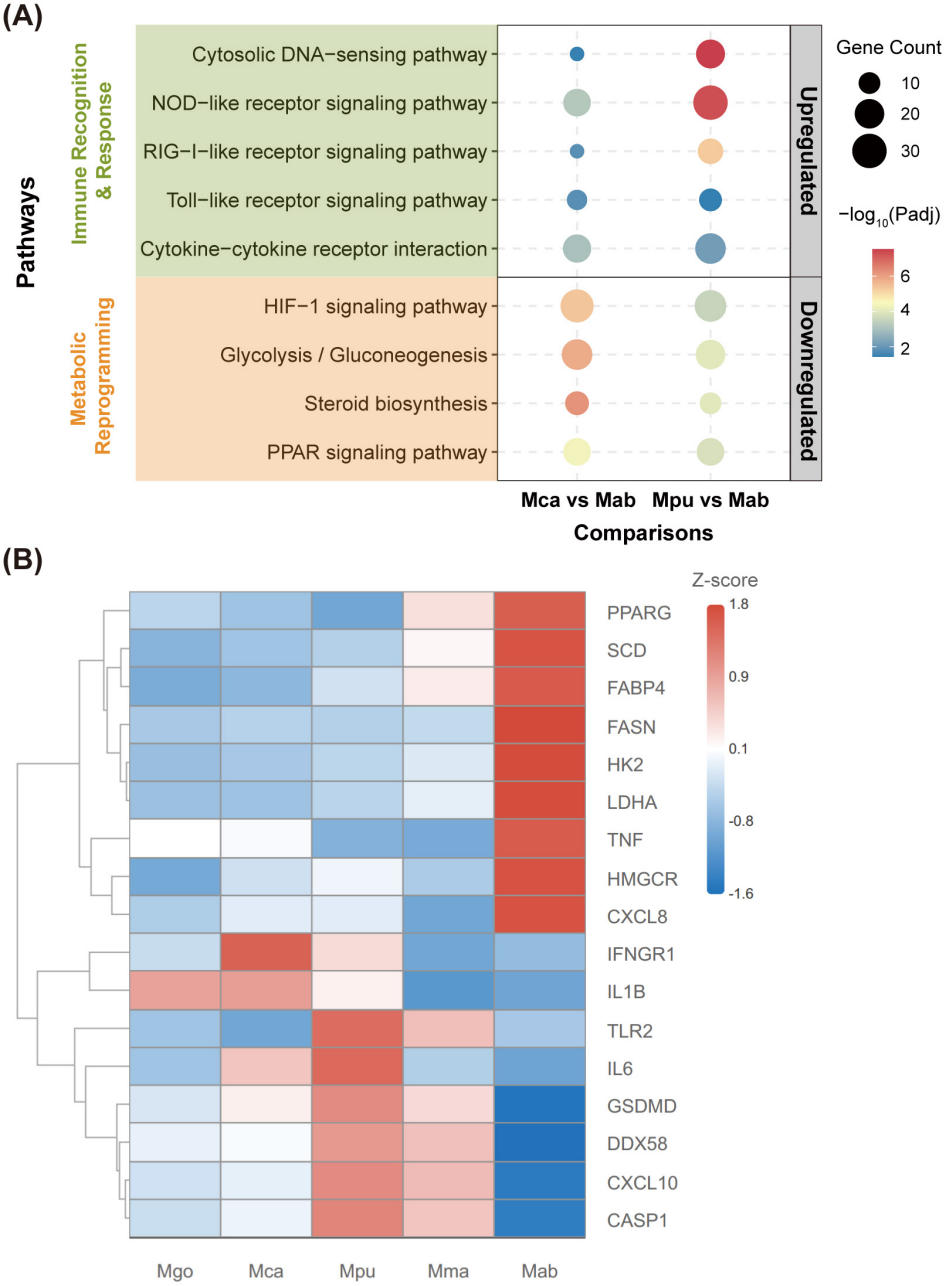
KEGG pathway enrichment and immune-metabolic gene expression in infected macrophages. **(A)** Bubble plot of KEGG pathway enrichment for DEGs in THP-1–derived macrophages at 24 hpi. Pathways are grouped into immune recognition and response (green background) and metabolic reprogramming (orange background). Comparisons shown are *M. camsae* vs. *M. abscessus* (*Mca* vs *Mab*) and *M. pumcae* vs. *M. abscessus* (*Mpu* vs *Mab*). Bubble size represents the number of genes in each pathway, and color intensity indicates statistical significance (–log_10_ padj). Right-hand labels indicate whether pathways are upregulated or downregulated relative to Mab infection. **(B)** Heatmap showing expression of selected immune and metabolic genes in macrophages infected with different mycobacterial strains. Expression levels are displayed as *z*-scores calculated from normalized read counts, with red indicating higher expression and blue indicating lower expression. Genes were clustered using hierarchical clustering with Euclidean distance. Each column represents infection with a different strain. *Mab, M. abscessus*; *Mma, M. marinum*; *Mpu, M. pumcae*; *Mca, M. camsae*; *Mgo, M. gordonae*.

To interpret the dominant contrasts that emerged in comparative DEG analyses, we performed KEGG enrichment focusing on the differences between each novel species and the rapid-growing reference *M. abscessus* (Figure 5A). Enriched pathways mapped to two overarching themes: innate immune recognition/response and metabolic reprogramming. At 24 hpi, pathways related to innate sensing and cytokine signaling—including cytosolic DNA-sensing, NOD-like receptor, RIG-I-like receptor, and Toll-like receptor signaling—were significantly enriched among genes upregulated in macrophages infected with *M. camsae* or *M. pumcae* relative to *M. abscessus*. This indicates that at this time point, the host response to the novel slow growers is characterized by a comparatively stronger engagement of immune recognition modules. Conversely, metabolic pathways such as HIF-1 signaling, glycolysis/gluconeogenesis, steroid biosynthesis, and PPAR signaling were significantly downregulated in the novel-species infections compared to the *M. abscessus* condition, reflecting a more prominent metabolic remodeling signature induced by the rapid-grower.

The divergence between immune and metabolic states was further supported by the expression patterns of representative marker genes (Figure 5B). Metabolic markers associated with glycolysis (*HK2, LDHA*), lipid/cholesterol metabolism (*FASN, SCD, HMGCR*), and PPAR signaling (*PPARG, FABP4*) exhibited markedly higher expression in *M. abscessus* infections. In contrast, genes involved in immune recognition and effector functions—including pattern recognition receptors (*TLR2, DDX58*), inflammasome components (*CASP1, GSDMD*), and key cytokines/chemokines (*IL1B, IL6, CXCL10*)—showed coordinated elevation in the novel species, particularly in *M. pumcae*. Notably, *M. pumcae* induced higher levels of several immune markers than *M. camsae* and *M. gordonae*, consistent with its more robust stimulatory phenotype. Collectively, these results suggest that macrophages infected with the novel slow growers and those infected with the rapid-growing M. abscessus occupy distinct immune–metabolic transcriptional states at 24 hpi.

## 3 Discussion

The discovery of *M. pumcae* and *M. camsae* expands our understanding of the *M. gordonae* complex, moving beyond the traditional view of these organisms as mere environmental contaminants. Although these novel SGM are genomically similar to *M. gordonae*, they display distinct transcriptional programs and host–interaction strategies that shape their pathogenic potential.

A central finding of this work is the clear divergence in host-response strategies between SGM and the rapid-growing *M. abscessus*. At 24 hpi, macrophages infected with *M. abscessus* exhibited profound metabolic reprogramming, including activation of HIF-1α signaling, glycolysis and lipid biosynthesis, consistent with the high nutrient demand and aggressive replication strategy of rapid-growing mycobacteria (Kumar et al., 2025; Rizvi et al., 2019; Dong et al., 2025). In contrast, macrophages infected with the novel SGM showed preferential activation of innate immune-sensing pathways, with significantly stronger induction of cytosolic DNA-sensing, NOD-like receptor and Toll-like receptor signaling. This pattern suggests that SGM, by virtue of their slower growth, permit more sustained engagement of host pattern-recognition machinery, favoring the establishment of relatively stable intracellular niches rather than rapid host-cell lysis (Watson et al., 2012; Gopalakrishnan and Salgame, 2016; Dubé and Behr, 2023).

Among the two novel species, *M. pumcae* showed markedly greater intracellular persistence than *M. camsae*. Our bacterial transcriptomic data provide a molecular basis for this phenotype. *M. pumcae* exhibited elevated expression of the *dosR* and *mprA* regulons, which in *M. tuberculosis* are essential for sensing environmental stress and entering a persistent, non-replicative state within phagosomes (Doddam et al., 2019; Mehra et al., 2015; Bretl et al., 2012). Together with increased expression of iron acquisition and storage genes such as *mbtD* and *bfrA, M. pumcae* appears transcriptionally “pre-adapted” to the nutrient-limited and oxidative milieu of macrophages (Rodriguez, 2006; Parida et al., 2023; Wu et al., 2022). This pre-conditioning likely underlies its higher intracellular burden and the broader host transcriptional response compared with *M. camsae*.

The transcriptional differences observed between *M. pumcae* and *M. camsae* in this study are consistent with and extend our previous genomic characterization of these species (Mei et al., 2025). In our genome-wide annotation, comparative analysis using OrthoVenn3 revealed that *M. pumcae* (Z3061^T^) and *M. camsae* (X7091^T^) shared 3,696 core gene clusters (81%), while each species possessed a distinct set of species-specific genes: *M. camsae* harbored 884 unique genes enriched in pathogenesis, cell surface, and plasma membrane functions, whereas *M. pumcae* contained 539 unique genes enriched in protein serine/threonine kinase activity and cell wall organization. The transcriptomic data presented here reflect these underlying genomic differences. *M. camsae* showed elevated expression of cell envelope biosynthesis genes (*hadA, hadB, hadC, kasA, kasB*), consistent with its genomic investment in cell surface and membrane-related functions. *M. pumcae*, on the other hand, exhibited higher baseline expression of stress-response regulators (*dosR, mprA*), oxidative defense enzymes (*sodC*), and iron acquisition systems (*mbtD, bfrA*), which aligns with its genomic enrichment in kinase signaling and cell wall remodeling pathways. These convergent lines of evidence from genomics and transcriptomics suggest that the divergent pathogenic potential of these two species is shaped not only by differential gene regulation but also by the distinct genomic architectures that define each species' functional repertoire.

These findings also have important clinical implications. *M. gordonae* is frequently recovered from clinical specimens yet is often dismissed as a contaminant because of its perceived low virulence (Bittner and Preheim, 2016). Our data indicate that members of the *M. gordonae* complex, particularly *M. pumcae*, harbor genetic and transcriptional modules that support prolonged intracellular survival. The distinct host transcriptional signatures we observed—with *M. pumcae* inducing substantially more up-regulated host genes than *M. gordonae*—argue that these species are not functionally redundant and should not be uniformly regarded as innocuous.

This study has several limitations that should be considered when interpreting the results. First, THP-1-derived macrophages, while widely used as a standardized infection model, do not fully replicate the complexity of primary human macrophages or the *in vivo* skin microenvironment where these organisms cause infection. Future validation using primary macrophages or 3D skin models is warranted. Second, a uniform MOI of 10:1 was applied across all strains despite their vastly different growth rates. The higher bacterial burden of *M. abscessus* at later time points likely reflects a combination of intracellular replication and extracellular growth following macrophage lysis. Third, the host RNA-seq analysis was restricted to a single time point (24 hpi). Although this snapshot revealed clear differences between slow-growing mycobacteria and *M. abscessus*, it does not capture the temporal evolution of the host response, such as early innate sensing (2–6 hpi) vs. later immunometabolic adaptation (48–72 hpi). Fourth, the prokaryotic transcriptomes were obtained from bacteria grown in axenic culture, rather than from intracellular organisms. The observed expression patterns should therefore be interpreted as species-specific pre-adaptation signatures rather than direct intracellular responses. Future work combining multiple infection time points with dual RNA-seq of host cells and intracellular bacteria, as well as integration with proteomic and metabolomic data, will be crucial to delineate the dynamic and reciprocal regulation between *M. pumcae, M. camsae* and their host.

In conclusion, our study shows that the pathogenic potential of non-tuberculous mycobacteria is determined by a balance between bacterial stress-response capacity and host immunometabolic signaling. *M. pumcae* represents a distinct evolutionary branch within the *M. gordonae* complex that has optimized persistence through *dosR*/*mprA*-dependent pathways, eliciting a stronger host response than its close relatives. These results support a more nuanced clinical approach in which precise genomic identification of non-tuberculous mycobacteria is integrated into diagnosis and risk stratification to distinguish true environmental contaminants from opportunistic pathogens.

## 4 Materials and methods

### 4.1 Bacterial strains and culture conditions

Five mycobacterial strains were used in this study: *Mycobacterium camsae* X7091^T^ (= CGMCC 1.90336^T^ = JCM 37414^T^), *Mycobacterium pumcae* Z3061^T^ (= CGMCC 1.90337^T^ = JCM 37415^T^), *Mycobacterium gordonae* DSM 44160^T^, *Mycobacterium marinum* ATCC 927^T^, and Mycobacteroides abscessus ATCC 19977^T^. Bacterial strains were routinely cultured in Middlebrook 7H9 broth (BD Difco) supplemented with 10% oleic acid-albumin-dextrose-catalase (OADC; BD BBL), 0.5% glycerol, and 0.05% Tween 80 (Solarbio) at 32 °C (*M. marinum*) or 37 °C (all other strains) with shaking at 100 rpm.

### 4.2 Growth curve determination

For growth curve analysis, bacterial cultures were adjusted to an initial OD600 of 0.05 in 50 mL of supplemented 7H9 medium and incubated at the appropriate temperature. Optical density measurements were recorded for 30 days using a spectrophotometer (Thermo Scientific). Three independent experiments were performed for each strain.

### 4.3 Cell culture and macrophage differentiation

The human monocytic cell line THP-1 (ATCC TIB-202) was maintained in RPMI 1640 medium (Gibco) supplemented with 10% fetal bovine serum (FBS; Gibco), 100 U/mL penicillin (Gibco), and 100 μg/mL streptomycin (Gibco) at 37 °C in a humidified atmosphere containing 5% CO_2_. For macrophage differentiation, THP-1 cells were seeded in 6-well plates (8 × 10^5^ cells/well) and treated with 100 ng/mL phorbol 12-myristate 13-acetate (PMA; Sigma-Aldrich) for 48 h. Cells were then washed and rested in fresh complete medium without PMA for 24 h before infection.

### 4.4 Macrophage infection and intracellular survival assay

Bacterial cultures in mid-logarithmic phase were harvested, washed twice with PBS, and single-cell suspensions were prepared by passing through a 27-gauge needle. Differentiated THP-1 macrophages were infected at an MOI of 10:1 for 4 h at 37 °C. Extracellular bacteria were removed by washing three times with PBS. To eliminate any remaining extracellular and surface-adherent bacteria, infected macrophages were incubated with fresh medium containing 50 μg/mL gentamicin (Sigma-Aldrich) for 2 h at 37 °C. After gentamicin treatment, cells were washed three times with PBS and replenished with antibiotic-free medium for the remainder of the incubation period. At designated time points (0, 24, 48, and 72 hpi), cells were lysed with 0.1% Triton X-100 (Sigma-Aldrich), and serial dilutions were plated on Middlebrook 7H10 agar (BD Difco) supplemented with OADC. Colonies were counted after incubation at the appropriate temperature for 3–4 weeks (slow-growing mycobacteria) or 3–5 days (*M. abscessus*). Results are expressed as Log_10_CFU/well. Three independent experiments were performed.

### 4.5 LDH cytotoxicity assay

LDH release was measured using the Cytotoxicity LDH Assay Kit (MedChemExpress) according to the manufacturer's instructions. Culture supernatants were collected at 0, 24, 48, and 72 hpi from three independent biological replicates per group and assayed in duplicate. LDH release was calculated as a percentage of the maximum LDH release after subtracting the spontaneous LDH release from uninfected control cells.

### 4.6 Cytokine measurements

Culture supernatants were collected at 6, 24, and 48 hpi and stored at −80 °C until analysis. Concentrations of TNF-α, IL-6, and IL-1β were measured using commercial ELISA kits (R&D Systems) according to the manufacturer's instructions. All samples were assayed in duplicate, and three independent experiments were performed.

### 4.7 Phylogenetic analysis

16S rRNA gene sequences were aligned using MUSCLE, and a neighbor-joining phylogenetic tree was constructed using MEGA X software with 1,000 bootstrap replications. The scale bar represents 0.01 nucleotide substitutions per site (Kumar et al., 2018).

### 4.8 Total RNA Extraction

Total RNA was extracted from two experimental sets: bacteria grown in Middlebrook 7H9 liquid medium (logarithmic phase) and macrophages infected with different mycobacterial strains at 24 hpi. For the liquid culture samples, bacterial pellets were collected by centrifugation and resuspended in TRIzol reagent (Invitrogen). For the infection samples, infected macrophages were washed with PBS and lysed directly in TRIzol. To ensure efficient lysis of the mycobacterial cell wall, all samples were subjected to mechanical disruption using 0.1 mm silica beads in a high-speed cell crusher. Total RNA was then purified through a standard chloroform extraction and isopropanol precipitation protocol. The concentration, purity, and integrity of the isolated RNA were assessed using a NanoDrop 2000 spectrophotometer and an Agilent 2100 Bioanalyzer to ensure that the RNA integrity number (RIN) exceeded 7.0 for all samples used in subsequent applications.

### 4.9 Transcriptome sequencing

Two distinct library preparation strategies were implemented to accommodate the structural differences between eukaryotic and prokaryotic RNA. For the host eukaryotic transcriptome from infected macrophages, mRNA was specifically enriched using Oligo(dT) magnetic beads to capture polyadenylated transcripts. For the prokaryotic transcriptome of the novel species in liquid culture, ribosomal RNA (rRNA) was removed using a ribo-zero depletion kit (Illumina Stranded Total RNA Prep with Ribo-Zero Plus) to enrich the non-polyadenylated bacterial mRNA. After enrichment or depletion, the remaining RNA was fragmented into short templates for first- and second-strand cDNA synthesis. The resulting cDNA fragments underwent end-repair, A-tailing, and ligation with Illumina-indexed sequencing adapters. The finalized libraries were amplified by PCR and sequenced on the Illumina NovaSeq 6000 platform using a 150-bp paired-end sequencing strategy.

### 4.10 Bioinformatic analysis

The raw sequencing data were first processed with fastp to remove low-quality reads and adapter sequences. Gene expression levels were quantified as raw counts using featureCounts and subsequently normalized to Transcripts Per Million (TPM) for comparative analysis. Differential expression analysis was performed using the DESeq2 R package, where genes with an absolute Log_2_ fold change > 1 and an adjusted *p*-value < 0.05 were identified as DEGs (Love et al., 2014). Functional characterization was conducted through KEGG enrichment analyses using the clusterProfiler R package. Statistical visualizations, including heatmaps and pathway enrichment plots, were generated using the pheatmap and ggplot2 packages in the R environment. Detailed sequencing quality metrics, including total raw and clean read counts, library sequencing depth, Q20 and Q30 quality scores, GC content, reference genome mapping rates, and uniquely mapped read percentages for all libraries, are provided in Supplementary Table 1. The median-of-ratios method was applied for DESeq2 normalization, and the Benjamini-Hochberg FDR correction was used for multiple testing control. For the UpSet plots (Figures 4C, D), an exploratory threshold of raw *p*-value < 0.05 and |log_2_FC| > 1 was applied; all conclusion-level analyses, including KEGG pathway enrichment, are based on the formal DEG criteria (padj < 0.05 and |log_2_FC| > 1). Complete DEG lists are provided in Supplementary Table 2.

### 4.11 Statistical analysis

All quantitative data are presented as mean ± SEM from at least three independent experiments, unless otherwise stated. For cytokine concentration comparisons across multiple groups and time points, two-way analysis of variance (ANOVA) followed by Tukey's multiple comparisons test was performed using GraphPad Prism 10. For pairwise comparisons between two groups, unpaired two-tailed Student's *t*-test was applied. *P*-values < 0.05 were considered statistically significant. For the LDH cytotoxicity assay, one-way ANOVA with Tukey's *post hoc* test was used at each time point. Statistical details for each experiment, including the exact n value and statistical test applied, are reported in the corresponding figure legends.

## Data Availability

The raw data generated in this study can be found here: https://www.ncbi.nlm.nih.gov/geo, accession GSE328953 (eukaryotic/host transcriptomes) and GSE328954 (prokaryotic/bacterial transcriptomes).
