# Correction: Phenotypic and transcriptomic characterization of two newly described Mycobacterium species within the *Mycobacterium gordonae* complex

**DOI:** 10.3389/fmicb.2026.1894087

**Published:** 2026-06-11

**Authors:** Tian Gan, Youming Mei, Haiqin Jiang, Wenyue Zhang, Ying Shi, Hongsheng Wang

**Affiliations:** 1 Hospital for Skin Disease, Institute of Dermatology, Chinese Academy of Medical Sciences and Peking Union Medical College, Nanjing, China; 2 Jiangsu Key Laboratory of Molecular Biology for Skin Diseases and STIs, Nanjing, China

**Keywords:** immunometabolism, macrophage, *Mycobacterium gordonae* complex, non-tuberculous mycobacteria, transcriptome

**Affiliation** Hospital for Skin Disease, Institute of Dermatology, Chinese Academy of Medical Sciences and Peking Union Medical College, Nanjing, China was erroneously given as Institute of Dermatology, Chinese Academy of Medical Sciences and Peking Union Medical College, Nanjing, China.

The original version of this article has been updated.

